# Effects of dietary Docosahexaenoic, training and acute exercise on lipid mediators

**DOI:** 10.1186/s12970-016-0126-y

**Published:** 2016-04-05

**Authors:** X. Capó, M. Martorell, A. Sureda, J. A. Tur, A. Pons

**Affiliations:** Research Group on Community Nutrition and Oxidative Stress, Science Laboratory of Physical Activity, Department of Fundamental Biology and Health Sciences, University of Balearic Islands, Crtra. Valldemossa, km 7.5, E-07122 Palma de Mallorca, Illes Balears Spain; CIBER: CB12/03/30038 Pathophysiology of Obesity and Nutrition, CIBEROBN, Health Institute Carlos III (ISCIII), University of Balearic Islands, 07122 Palma de Mallorca, Spain; Department of Nutrition and Dietetics, Faculty of Pharmacy, University of Concepción, 4070386 Concepción, Chile

**Keywords:** Inflammation, Docosahexaenoic acid, PGE_1_, PGE_2_, RvD1, Lipid mediators, Exercise

## Abstract

**Background:**

Eicosanoids mediate initiation and resolution of inflammation. Our aim was evaluating the effects of training, exercise and docosahexaenoic (DHA) supplementation on plasma eicosanoids levels and peripheral blood mononuclear cells (PBMCs) eicosanoids production.

**Methods:**

Fifteen male footballers were distributed to placebo and experimental groups. Experimental group consumed DHA-enriched beverage (1.16 g DHA/day) for 8 weeks, placebo group consumed a placebo beverage. Blood samples were taken before and after the nutritional intervention in basal conditions and 2 h after acute exercise.

**Results:**

Training increased basal Prostaglandin E1 (PGE_1_) plasma levels and PBMCs cyclooxygenase 2 (COX-2) protein levels in both groups, but COX-1 protein levels only in the experimental group. Acute exercise increased plasma PGE_2_ and PBMCs active NFκβ levels. Lipopolysaccharide (LPS)-stimulated PBMCs increases eicosanoids production (PGE_1_, PGE_2_, RvD1) in both groups and increased LPS-stimulated PBMCs active NFκβ. DHA supplementation increased COX-2 levels but decreased LPS-stimulated PBMCs PGE_1_ and PGE_2_ production. Neither DHA supplementation nor acute exercise altered the expression of NFκβ, COX-2, 15-LOX2, 5-LOX, or IL-1β genes in PBMCs.

**Conclusions:**

The increase of PGE_1_ plasma levels after training promoted systemic anti-inflammatory and vasodilator environment. Exercise and DHA supplementation acted synergistically by increasing plasma PGE_2_ with anti-inflammatory effects. Exercise primed PBMCs to enhance PGE_1_, PGE_2_ and RvD1 production in response to LPS.

**Trial registration:**

The project was registered at ClinicalTrial.gov (NCT02177383).

## Background

The practice of regular physical exercise has many health benefits, reducing the risk of suffering from several diseases such as cardiovascular disease, cancer, diabetes and osteoporosis [25]. Exercise exerts anti-inflammatory effects mediated by reduction of visceral fat mass [25], but may also be related to decreased levels of Toll-Like Receptors (TLRs) in monocytes [12] and to muscle production of bioactive compounds during contraction [29]. Each bout of acute exercise results in alterations in the pro/anti-inflammatory cytokine balance [6, 7, 36]. Moreover, the increased production of anti-inflammatory cytokines during exercise counteracts the production of pro-inflammatory cytokines associated to muscle damage, resulting in the instauration of an anti-inflammatory environment [28]. In addition, acute exercise alters the number and function of circulating immune cells [36], which are primed for their extravasation into tissues after exercise, thereby contributing to repair the damaged tissue [19].

Polyunsaturated omega-3 fatty acids (ω-3 PUFA) contribute to regulate inflammation associated with chronic diseases including obesity, arthritis and atherosclerosis [4]. The anti-inflammatory effects of ω-3 PUFA are evidenced by the inhibition of endotoxin-stimulated production of IL6 and IL8 in endothelial cells [18]. However, ω-3 PUFA diet supplementation does not affect plasma cytokine response to strenuous exercise, which produces muscle damage and inflammation [6, 7, 36]. The anti-inflammatory effects of ω-3 PUFA are mediated by their participation in the regulation of the nuclear factor κβ (NFκβ) signalling pathway and/or peroxisome proliferator-activated receptor (PPARγ) [3, 13]. The expression of pro-inflammatory genes via the NFκβ signalling pathway decreases after twelve weeks of ω-3 PUFA diet supplementation [31]. ω-3 PUFA diet supplementation also alters the function of immune cells [3], modulating chemotaxis and leukotriene generation in neutrophils by reducing arachidonic acid (AA) concentrations [20]. Eicosapentaenoic acid and docosahexaenoic acid (DHA) can compete with AA as a substrate for cyclooxygenase 2 (COX-2) and 5-lipoxygenase (5-LOX), thus reducing the production of inflammatory mediators derived from AA. This process plays a critical role in the initial inflammatory response [6, 7, 37]. Prostaglandins (PGs) mediators arising from the COX-1 and COX-2 cascade from AA or its precursors are central to vascular responses, permitting neutrophils and monocytes to leave post capillary venules. PGs also play a role in the regulation of muscle protein metabolism [2], vasodilation, human skeletal muscle microcirculation, febrile and inflammatory responses [39], and initiation and timely resolution of inflammation [32]. Prostaglandin E1 (PGE_1_) and prostaglandin E2 (PGE_2_) are potent vasodilators which account for the increased blood flow in inflamed areas but PGE_1_ also regulates neutrophil function by reducing neutrophil activation [41]. Because of these vasodilator and immune properties, PGs are used as treatment for diseases derived from ischemia-reperfusion, such as arterial occlusive disease and venous ulcers [11, 22, 41].

The response of PGs to exercise has not been clearly established yet. Skeletal muscle produces PGs in response to muscular work, and this production is blocked by the intake of COX inhibitors, [22, 38]. Plasma PGE_2_ concentration increases in response to exercise or to muscle damage [1]. In other words, exercise increases PGE_2_ production as part of the inflammatory response, triggered by micro trauma occurring in skeletal muscles, [1]. In addition, PGE_2_ synthesis by infiltrating macrophages in the inflamed muscle increases 24–48 h after an exercise session [40]. It is also noticeable that downhill running for 45 min at 75 % VO_2_max increases circulating monocyte production of PGE_2_ [5]. The effects of PGE_2_ can be described as pro-inflammatory or anti-inflammatory depending on location, since PGE_2_ enhances LTB4-mediated neutrophil extravasation and tissue injury, but it can also inhibit the NFκβ signalling pathway in macrophages [21]. This inhibition plays a critical function in the initiation of resolution via lipid mediator class switching [32].

A new family of lipid mediators produced from the oxidation of ω-3 PUFA including resolvins (Rvs), maresins and protectins has been described as pro-resolving mediators of inflammation [33]. The synthesis of these products involves COX and LOX pathways from DHA and EPA [33]. Rvs have been reported to reduce inflammation in chronic inflammatory diseases. Specifically, RvE1 and RvD1 inhibit trans-endothelial migration of neutrophils, preventing the infiltration of neutrophils into sites of inflammation, and RvD1 also inhibits IL1β production [34]. Plasma RvE1 and RvD1 have also been demonstrated to increase after acute exercise [22] but the participation of these lipid mediators in the anti-inflammatory effects of acute exercise has not been studied.

The aim of this study was to evaluate the effects of regular training and DHA diet supplementation on plasma lipid mediators. The effects of DHA diet supplementation and acute exercise on plasma prostaglandins and on PBMC capabilities to produce cytokines, prostaglandins and pro-resolving mediators in response to lipopolysaccharide (LPS) stimulation were also analysed.

## Methods

### Subjects and anthropometric characteristics

Fifteen male soccer players from the Real Mallorca B team volunteered to participate in this study, 6 subjects took one liter of a placebo beverage five times a week and the other 9 an experimental beverage rich in DHA, over a period of 8 weeks. The subjects and study design was the same described previously [6, 7, 23, 24]. At the beginning of the study, 22 subjects were recruited, but 6 of them left the football team during the experimental time and joined the first and professional team and one broke the anterior cruciate ligament of the knee. All subjects gave their written informed consent after an explanation of the experimental procedures and before commencement of the study. The study protocol was in accordance with the Declaration of Helsinki for research on human subjects and was approved by the Ethical Committee of Clinical Investigation of the CAIB (Palma de Mallorca, Balearic Islands, Spain). The project was registered at ClinicalTrial.gov (NCT02177383).

Height was determined using a mobile anthropometer (Kawe 44444, Asperg, Germany) to the nearest millimetre, with the subject’s head in the Frankfurt plane. Body weight was determined to the nearest 100 g using a digital scale (Tefal, sc9210, Rumilly, France). The subjects were weighed in bare feet and light underwear, and the mean of three measurements was used [6, 7, 23, 24]. Body mass index was calculated using the following equation: [BMI = mass (kg)/squared height (m)]. Participants in the study were 19.7 ± 0.4 years old, 76.5 ± 2.5 kg weight, and 179.5 ± 2.5 cm height. The waist circumference was 78.4 ± 0.9 cm, hip circumference was 97.4 ± 1.2 cm, waist-hip ratio (WHR) was 0.81 ± 0.01. The systolic blood pressure 119.5 ± 6.5 mmHg, and 61.7 ± 4.7 mmHg for the diastolic blood pressure. The Body Mass Index (BMI) was 23.7 ± 0.55, kg/m^2^. The soccer players had 92.6 ± 0.2 % fat free mass. The VO_2_max determined following the test of Leger-Boucher, was 61.4 ± 1.35 mL/kg min. There were no significant differences between groups in the anthropometric characteristics of players.

### DHA supplementation

DHA was administered to the athletes using an almond-based isotonic beverage. Both placebo and supplemented beverages were made up of 3.0 % almond and 0.8 % sucrose and the rest was water, flavour, and added oils and vitamin E. Moreover, the placebo drink contained 0.8 % refined olive oil whereas the experimental drink contained 0.6 % refined olive oil and 0.2 % wt % DHA-S (DSM, Columbia, USA). The two almond beverages were manufactured by Liquats Vegetals S.A. (Girona, Spain) following an industrial process. Two beverages were made-up by: cinnamon and lemon natural flavours, sucrose, vitamin E, and the respective oil for the experimental (olive oil plus DHA-S) or placebo (olive oil) drink [6, 7, 23, 24]. Finally, the beverages were sterilized and packed. Both types of beverages were identical in taste and visual appearance. Beverages fatty acid composition was determined following the same procedure previously described [23, 24]. The experimental beverage had a significantly greater content of C20:3 (0.640 mg/100 ml of beverage), C22:0 (2.57 mg/100 ml of beverage), C22:5 (56.1 mg/100 ml of beverage) and C22:6n3 (114 mg/100 ml of beverage) compared to the placebo beverage, in which these fatty acids were not present. No significant differences between beverages were evidenced in vitamin E content (41.6 mg/L of beverage n placebo drink, and 45.7 mg/L of beverage in the experimental drink).

### Dietary intake and nutritional intervention

Dietary habits of subjects were assessed using a 7-day dietary record questionnaire completed at the beginning of the study and in the week before the exercise test as previously described [6, 7, 23, 24]. A qualified dietician verified and quantified the food records. All food items consumed were transformed into nutrients using a special computerized program based on the European and Spanish food composition tables [6, 7, 23, 24]. DHA intake in the placebo group from the diet was 109 ± 40.7 mg/day, and DHA intake in the experimental group was 1209 ± 40.7 mg/day. The five times a week intake of 1 L of the supplemented beverage supposed a daily intake of about 1.14 g of DHA additional to the basal DHA intake through the diet in the experimental group, whereas the placebo only took up DHA from the diet.

This nutritional intervention with the respective beverages for eight weeks is reflected in the erythrocytes fatty acid composition [23, 24].

### Experimental procedure

For each subject two blood samples were taken at the end of the nutritional intervention, in basal conditions and after performing a soccer training session. The exercise consisted in a 2 h of habitual physical training session. After a 15 min warm-up, the players performed the Leger Boucher test. After that, players practiced a recovery exercise of control-passing for 15 min. The main body of the training session was characterized by small-sided games. Briefly, the first exercise consists of 5 vs 5 possession exercise in an area of 20 x15 m (4 repetitions of 5 min with 30 s of recovery between repetitions); the second was a 6 vs 6 possession exercise in an area of 30 x 20 m (3 repetitions of 6 min with 1 min of recovery between repetitions), and finally, the players played a soccer match 5 vs 5 in 30 x 40 m for 20 min. The training session was designed to perform exercise intensity at more than 70 % VO_2_max over 50 % duration of the session in order to induce an oxidative stress situation [35]. These conditions were followed by all participants in the study.

Blood samples used for analysis were collected from the same athletes, at the same time, using the same exercise protocols as previous described [6, 7, 23, 24]. The blood samples used to measure prostaglandins and Rvs plasma levels, cytokine production and expression were the same as those previously used [6, 7].

### PBMCs purification

Blood samples were centrifuged at 900 g at 4 °C for 30 min and the plasma was keep. The erythrocyte phase at the bottom was washed with PBS, centrifuged as above and finally erythrocytes were reconstituted with distilled water. The PBMCs fraction was purified from whole blood following an adaptation of the method previously described [6, 7] using Ficoll-Paque PLUS reagent (GE Healthcare®). An aliquot of PBMCs was used for LPS incubation.

### PBMC incubation with LPS

Incubations of PBMCs were performed in RPMI 1640 culture media containing 2 mM l-glutamine in the presence of the bacterial stimuli LPS. PBMCs obtained after 8-week beverage supplementation in basal conditions and after exercise were diluted with RPMI 1640 culture media to 2x10^6^ cells/ml and activated by addition of LPS from *Escherichia coli* (1 μg/ml). Samples were incubated in polypropylene tubes at 37 °C for 2 h. In a parallel experiment, an aliquot of PBMCs obtained after exercise were activated with LPS and incubated at either 37 °C or 39.5 °C for 2 h. Then, after shaking, the cells from both experiments were pelleted by centrifugation (900 g, 5 min) and the cell-free supernatants were stored at -70 °C for cytokine determinations.

### Active NFκβ determination

An isolated suspension of PBMCs was subjected to whole-cell protein extraction for the determination of NFκβ p50 activation, which was performed using the ELISA method TransAM NF-kB p50 Chemi according to the manufacturer’s instructions (Active Motif®). Briefly, the primary antibody used to detect NFκβ recognizes an epitope on p50 that is accessible only when NFκβ is activated and bound to its DNA target.

### Cytokine and lipid mediator determination

IL1β and MCP1 were measured in culture medium supernatant using ELISA kits. IL1β and MCP1 kits (RayBio®); intra-assay and inter-assay reproducibility for both kits were lower than 10 % and 12 %.

PGE_1_ and PGE_2_ were measured in plasma and in culture medium supernatant using ELISA kits (Enzo Life Sciences®). Intra-assay and inter-assay reproducibility for PGE_1_ were lower than 10 % and 12 %, respectively, while intra-assay and inter-assay reproducibility for PGE_2_ were lower than 6 % in both cases.

RvD1 concentration in culture medium supernatants was determined using an RvD1 EIA Kit (Cayman®), following the instructions manual. Intra-assay reproducibility was 10 %.

### PBMCs RNA extraction and real time PCR assay

COX-2, NFκβ, 15-LOX2, IL-1β, 5-LOX mRNA levels were determined by multiplex real time PCR based on incorporation of a fluorescent reporter dye and using human 18S rRNA as reference. For this purpose, total RNA was isolated from PBMCs by Tripure extraction (Roche Diagnostics®). RNA (1 μg) from each sample was reverse transcribed using 50 U of Expand Reverse Transcriptase (Roche Diagnostics, Germany) and 20 pmol oligo for 60 min at 37 °C in a 10 μL final volume, according to manufacturer instructions. The resulting cDNA (2.5 μL) was amplified using the Light-Cycler FastStart DNA MasterPLUS SYBR Green I kit (Roche Diagnostics®). Amplification was performed at 55 °C and 45 cycles. The relative quantification was performed by standard calculations considering 2(-ΔΔCt). Inflammatory gene expression levels before and after the session were normalized to the invariant control 18S rRNA. mRNA levels at the beginning of the stage were arbitrarily referred to as 1. Primers used are 18S forward (Fw): 5′-ATG TGA AGT CAC TGT GCC AG-3′ and Reverse: 5′-GTG TAA TCC GTC TCC ACA GA-3′ annealing temperature 60 °C; COX-2 Fw: 5-TTG CTG GCA GGG TTG CTG GTG GTA-3′, and Rv: 5′-CAT CTG CCT GCT CTG GTC AAT GGA A-3′ annealing temperature 67 °C; NFκβ, Fw: 5′-AAA CAC TGT GAG GAT GGG ATC TG-3′ Rv: 5′-CGA AGC CGA CCA CCA TGT-3′ annealing temperature 60 °C; 15-LOX2, Fw: 5′-GCA TCC ACT GAT TGG ACC TT-3′ and Rv: 5′-GCT GGC CTT GAA CTT CTG AC-3′ annealing temperature 61 °C; IL-1β Fw: 5′-GGA CAG GAT ATG GAG CAA CA-3′, and Rv: 5′-GGC AGA CTC AAA TTC CAG CT -3′ annealing temperature 58 °C; 5-LOX, Fw: 5′-GGG CAT GGA GAG CAA AGA AG-3′ and Rv: 5′-ACC TCG GCC GTG AAC GT-3′ annealing temperature 59 °C.

### SDS-polyacrylamide gel electrophoresis and western blot analysis

Cells were lysed with 250 μL of RIPA buffer [250 mM Tris/HCl, pH 8.0, 4.4 % NaCl, 5 % IGEPAL®, 2.5 % deoxycholic acid, 0.5 % sodium dodecylsulfate (SDS)]. 20 μg of proteins of total cell extract was loaded on 12 % polyacrylamide gel, and were separated by size using SDS polyacrylamide gel. Following electrophoresis, samples were transferred onto a nitrocellulose membrane and incubated with a primary monoclonal antibody anti-COX-1 (1:1000) and anti-COX-2 (1:1000) (Cayman®).

Blots were then incubated with a secondary peroxidase-conjugated antibody (1:10,000), and the primary antibody was performed. Protein bands were visualized by Immun-Star® Western C® Kit reagent (Bio-Rad Laboratories) Western blotting detection systems. The chemiluminescence signal was captured with a Chemidoc XRS densitometer (Bio-Rad Laboratories®) and analyzed with Quantity One-1D Software (Bio-Rad Laboratories®).

### Statistical analysis

Statistical analysis was carried out using Statistical Package for Social Sciences (SPSS v.15.0 for Windows). Results are expressed as mean ± SEM. and *P* < 0.05 was considered statistically significant. A Kolmogorov-Smirnov test was applied to assess the normal distribution of the data. The statistical significance of the data was assessed by two-way analysis of variance (ANOVA). The statistical factors analysed were beverage supplementation (S), acute exercise (E), training season (T) and LPS stimulation (A). The data sets with a significant SxE, SxT, ExA, SxA and SxAxE interactions were tested by the ANOVA one-way test in order to identify groups who were different. When significant effects of S, E, T or A factor were found, a Student’s t test for paired data was used to determine the differences between the groups involved.

## Results

Normality of the data was assessed using the Kolmogorov-Smirnov test and the dependent variable was normally distributed in each group that was compared in the ANOVA test.

### Regular training and DHA diet supplementation effects

Training season and DHA diet supplementation significantly influenced PBMC counts. The training season increased circulating PBMCs from 2.79 ± 0.16x10^3^ to 3.34 ± 0.15x10^3^ cells/μL in the placebo group, and from 3.23 ± 0.29x10^3^ to 3.99 ± 0.34x10^3^cells/μL in the experimental group. This increase only was significantly in the experimental group.

DHA diet supplementation for 8 weeks caused a variation in the DHA percentage on erythrocytes membrane (results not shown). The initial percentage of DHA respect to erythrocyte total fatty acid in placebo group (7.37 ± 0.27 %) was maintained after the diet supplementation with placebo beverage (8.40 ± 0.41 %), whereas the initial percentage of DHA in experimental group (7.93 ± 0.54 %) was significantly increased (10.8 ± 0.50 %) to attain a significantly higher content than the placebo group.

**Fig. 1:**
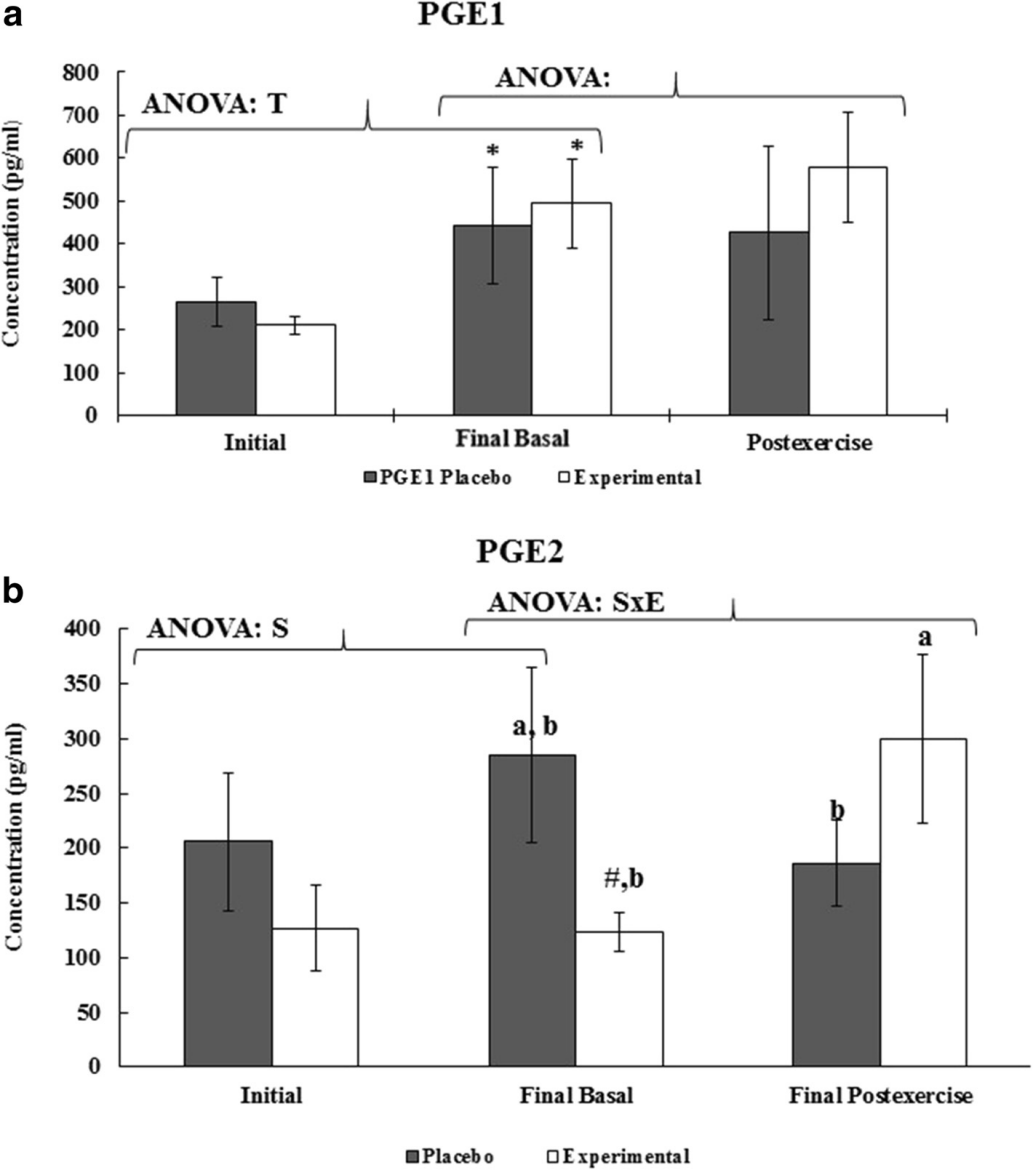
Effects of acute exercise, DHA diet supplementation and training season on PGE1 and PGE2 plasma levels. Statistical analysis: Two-way ANOVA, *p* < 0.05. (T) Significant effect of Training season, (S) Significant effect of Supplementation, (E) Significant effect of Acute Exercise, (ExT) Significant interaction between both factors. One-way ANOVA, *p* < 0.05. (*) Significant difference between Initial and Final, (#). Significant differences between Placebo and Experimental groups, ($) Significant differences between the Basal and Post-exercise. When interaction exists between different groups, different letters reveal significant differences. Results are the mean ± SEM

Regular training of soccer players for 8 weeks significantly influenced the basal plasma levels of PGE_1_ whereas DHA diet supplementation significantly changed the basal plasma levels of PGE_2_ (Fig. 1). Plasma PGE_1_ significantly increased after 8 weeks of training in both placebo and experimental groups, while plasma PGE_2_ maintained the initial basal levels. No significant differences were reported in PGE_2_ at the beginning of the intervention although its levels in the experimental group were lower than in the placebo. This trend was magnified after 8 weeks training reporting significant differences due to DHA diet supplementation.

**Fig. 2:**
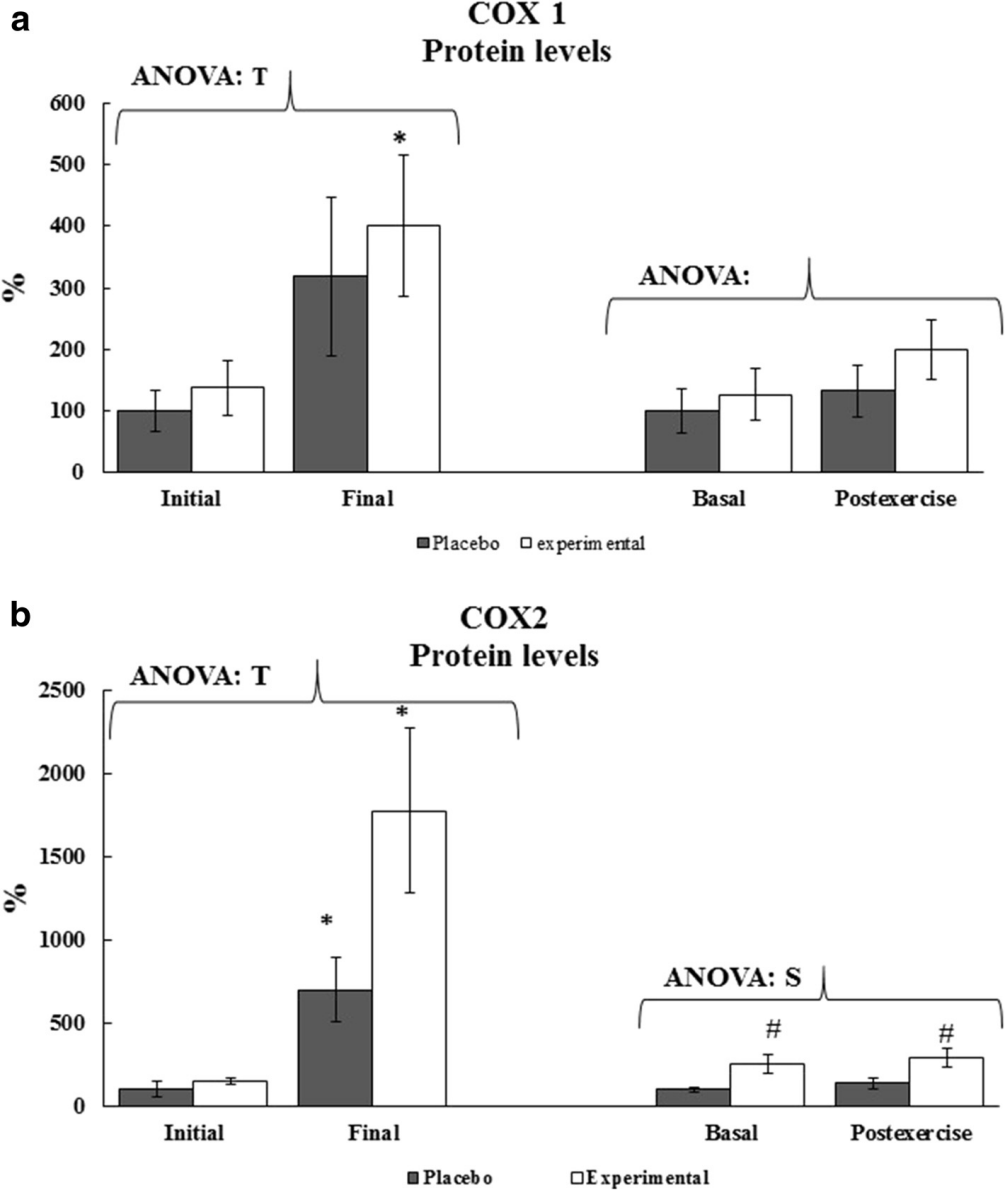
Effects of acute exercise, DHA diet supplementation and training season on COX1 and COX2 protein levels. Statistical analysis: Two-way ANOVA, *p* < 0.05. (T) Significant effect of Training season, (S) Significant effect of Supplementation, (E) Significant effect of Acute Exercise, (ExT) Significant interaction between both factors. One-way ANOVA, *p* < 0.05. (*) Significant difference between Initial and Final, (#). Significant differences between Placebo and Experimental groups, ($) Significant differences between the Basal and Post-exercise. When interaction exists between different groups, different letters reveal significant differences. Results are the mean ± SEM

Regular training but not DHA diet supplementation significantly influenced COX-1 and COX-2 protein levels in PBMCs (Fig. 2). Eight weeks of regular training significantly increased COX-1 and COX-2 protein levels in experimental group but not in placebo group.

### Acute exercise and DHA diet supplementation effects

Acute exercise did not influence PBMC counts, maintaining the basal values both in placebo and experimental groups (Placebo group: 3.34 ± 0.15 in basal conditions, 3.19 ± 0.15 after acute exercise; Experimental group: 3.99 ± 0.34 in basal conditions, 3.13 ± 0.16 after acute exercise, all PBMC results are expressed as 10^3^cells/μL).

The effects of acute exercise and DHA diet supplementation on PGE_1_ and PGE_2_ plasma levels are shown in Fig. 1. Acute exercise and DHA diet supplementation did not influence PGE_1_ plasma levels, whereas a statistically significant interaction between acute exercise and DHA diet supplementation was reported in plasma levels of PGE_2_. The experimental group showed significantly increased levels (about 2.4 times) of plasma PGE_2_ after acute exercise, whereas the placebo group maintained basal levels. This response resulted in higher PGE_2_ plasma levels in the experimental group (about 1.6 times) with respect to the placebo one.

The effects of DHA diet supplementation and acute exercise on the production rates of lipid mediators by LPS-stimulated PBMCs at 39.5 °C were also evaluated (Table 1). Acute exercise caused a significant increase in PGE_1_ and PGE_2_ production by LPS-stimulated PBMCs both in placebo and experimental groups. DHA diet supplementation significantly ameliorated the increase in both PGs production rate by LPS-stimulated PBMCs. The RvD1 production rate by LPS-stimulated PBMCs at 39.5 °C increased after acute exercise in both groups, but the increase was only significant (about 2.2 times) in the experimental group,

**Table 1:** Effects of acute exercise and DHA diet supplementation on lipid mediator PBMCs-stimulated production rates

		Basal	Post-exercise	ANOVA			
				S	E	SxE	Fd
PGE1 (pg/h 10^6^ cells)	Placebo	51,3 ± 8,19	159 ± 25^b^	.000 (*F* = 12.32)	.002 (*F* = 25.786)	.102 (*F* = 2.917)	(3, 22)
	Experimental	22,6 ± 6,47	76,2 ± 11,1^ba^				
PGE2 (pg/h 10^6^ cells)	Placebo	263 ± 51,7	615 ± 114^a^	.000 (*F* = 11.07)	.003 (*F* = 22.787)	.813 *(F* = .057)	(3, 22)
	Experimental	46,5 ± 14,7^b^	365 ± 40,5^ab^				
RvD1 (pg/h 10^6^ cells)	Placebo	4.24 ± 0.74	7.24 ± 1,69	.956 (*F* = .003)	.007 (*F* = 8.949)	.0668 (*F* = .189)	(3, 22)
	Experimental	3.79 ± 0,39	7.81 ± 1,11^b^				

Statistical analysis: Two-way ANOVA, *p* < 0.05., (S) Significant effect of Supplementation, (E) Significant effect of Acute Exercise, (ExS) Significant interaction between both factors. One-way ANOVA, *p* < 0.05. (^a^) Significant differences between Placebo and Experimental groups, (^b^) Significant differences between Basal and Post-exercise. When interaction exists between different groups, different letters reveal significant differences. Results are the mean ± SEM. Fd means Freedom degree

The effects of LPS-stimulation of PBMCs, acute exercise, and DHA diet supplementation on PBMC cytokine production are reported in Table 2. LPS-stimulation of PBMCs significantly increased IL1β basal production rate in both placebo (about 2.2 times) and experimental (about 1.8 times) groups, without any effect by DHA diet supplementation or acute exercise. No significant effects induced by LPS-stimulation or DHA diet supplementation were observed in MCP1 production rate. However, acute exercise significantly increased the MCP1 production rate by non-stimulated PBMCs, mainly in the experimental group; while no significant effects between groups of DHA diet supplementation were observed.

**Table 2:** Effects of LPS-stimulation, DHA diet supplementation and exercise on cytokine production rate by PBMCs

		Basal		Post-exercise		ANOVA
		NO LPS	LPS	NO LPS	LPS	
IL1β (pg/h 10^6^ cells)	Placebo	6.22 ± 1.46	16.3 ± 4.79$	6.68 ± 1.51	21.1 ± 7.34$	A (.002)
						(*F = 10.673*)
	Experimental	9.35 ± 2.24	17.6 ± 6.28$	8.34 ± 2.19	21.7 ± 8.07$	Fd (7,41)
MCP1 (pg/h 10^6^ cells)	Placebo	32.1 ± 9.79	36.1 ± 4.07	36.2 ± 6.14	45.4 ± 3.87	E(.002)
						(*F = 10.437*)
	Experimental	22.7 ± 5.31	29.6 ± 4.98	49.6 ± 4.73#	38.3 ± 3.81	Fd (7,41)

Statistical analysis: Two-way ANOVA, *p* < 0.05. S, Supplementation effect; A, LPS-stimulation effect, E, Exercise effect, SxA, interaction between Supplementation and LPS-stimulation effects, SxE, interaction between Supplementation and Exercise effects, ExA, interaction between Exercise and LPS activation effects, AxExS, interaction between effects of three factors. *Differences between Placebo and experimental groups; #difference between Basal and Post-exercise conditions p; $differences between NO LPS-stimulated and LPS-stimulated groups. When interaction exists between different groups, different letters reveal significant differences. Results are the mean ± SEM. Fd means Freedom degree

**Fig. 3:**
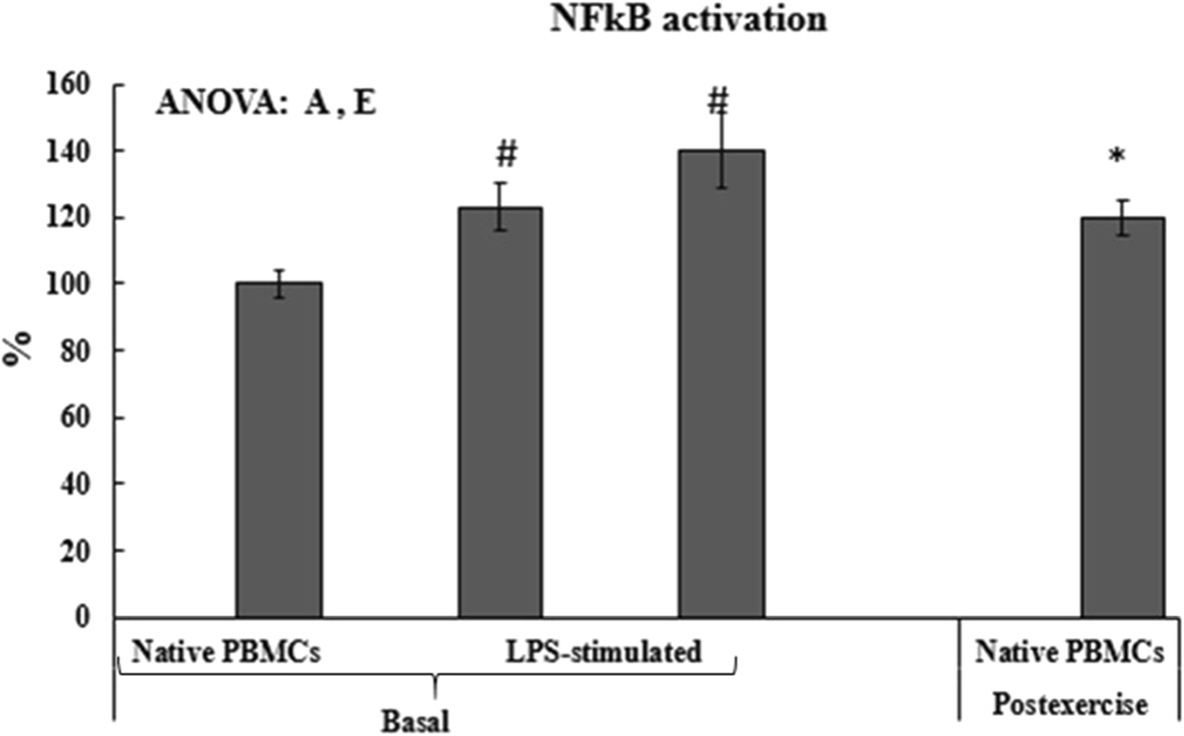
Effects of LPS stimulation, temperature and acute exercise on NFκβ activation in PBMCs. Statistical analysis: Two-way ANOVA, *p* < 0.05. (E) Significant effect of acute exercise, (A) Significant effect of LPS activation. One-way ANOVA, *p* < 0.05. (*) Significant differences between Basal and Post-exercise, (#) significant differences between No-stimulated PBMCs and LPS-stimulated PBMCs. When interaction exists between different factors, different letters reveal significant differences between groups. Results are the mean ± SEM

In order to evaluate the participation of NFκβ activation in the response to LPS-stimulation and to acute exercise, active NFκβ levels were determined in PBMCs (Fig. 3). Active NFκβ levels significantly increased about 20 % respect to the basal value after acute exercise. Basal PBMC incubation with LPS induced a significant increase of 27 % and 35 % in NFκβ activation at 37 °C and 39.5 °C, respectively.

The effects of acute exercise and DHA diet supplementation on the expression of inflammatory and anti-inflammatory genes, and COX-1 and COX-2 protein levels in PBMCs were determined (Table 3). No significant changes were observed in the expression of any of the evaluated genes due to acute exercise or DHA diet supplementation. Neither acute exercise nor DHA diet supplementation altered COX-1 protein levels in PBMCs (Fig. 2). COX-2 protein levels in PBMCs of the experimental group were significantly higher (about 2 times) than in the placebo group in both basal and post-exercise conditions; although no effects were evidenced due to the acute exercise.

**Table 3:** Effects of DHA diet supplementation and exercise on PBMCs gene expression

		Basal	Post-exercise	ANOVA			
				S	E	SxE	Fd
NFκβ (AU)	Placebo	1.00 ± 0.22	1.21 ± 0.31	.576 (*f* = .373)	.369 (*f* = .142)	.906 (*f* = .006)	(3,42)
	Experimental	1.33 ± 0.33	1.46 ± 0.33				
COX-2 (AU)	Placebo	1.00 ± 0.28	1.99 ± 0.86	.355 (*f* = .026)	.726 (*f* = .592)	.513 (*f* = .142)	(3,42)
	Experimental	1.46 ± 0.45	1.80 ± 0.51				
15-LOX2 (AU)	Placebo	1.00 ± 0.37	1.43 ± 0.76	.402 (*f* = .717)	.973 (*f* = .001)	.983 (*f* = .000)	(3,45)
	Experimental	1.03 ± 0.30	1.43 ± 0.43				
5-LOX (AU)	Placebo	1.00 ± 0.28	1.10 ± 0.42	.694 (*f* = .109)	.742 (*f* = .157)	.956 (*f* = .003)	(3,52)
	Experimental	0.89 ± 0.21	1.02 ± 0.18				
IL1β (AU)	Placebo	1.00 ± 0.34	0.84 ± 0.17	.906 (*f* = .000)	.998 (*f* = .014)	.496 (*f* = .472)	(3,42)
	Experimental	0.80 ± 0.22	1.03 ± 0.29				

Statistical analysis: Two-way ANOVA. (S) Significant effect of supplementation, (E) Significant effect of exercise, (SxE) Significant interaction between both factors. (#)Significant differences between Placebo and Experimental groups. (*)Significant differences between the Pre-exercise and Post-exercise. *p* < 0.05. When interaction exists between different groups, different letters reveal significant differences. Results are the mean ± SEM. Fd means Freedom degree. AU means arbitrary units referred to Pre-exercise Placebo values

## Discussion

### Effects on plasma prostaglandin levels

PGs are relatively transient molecules with a half-life of only seconds to minutes, and they work in an autocrine and paracrine approach through specific receptors [38]. Despite this signalling role and short half-life, basal plasma levels of PGE_1_ and PGE_2_ observed in soccer players are relatively high. Both molecules show similar values to those of untrained male university students [16], higher than those of ironman sportsmen [1], and lower than post-menopausal or young women [9]. Regular training for 8 weeks practically duplicated the basal plasma levels of PGE_1_, but did not affect basal plasma levels of PGE_2_. Treatments with PEG_1_ are used to ameliorate several diseases related to ischemia-reperfusion [11]. In these studies, PGE_1_ treatment induced vasodilatation, inhibited platelet and leukocyte aggregation and decreased oedema formation during reperfusion. The dose of PGE_1_ used in these treatments increased plasma PGE1 levels up to values similar to the basal plasma PGE_1_ levels observed in soccer players after a training season. The anti-inflammatory and health effects of regular exercise [25, 29] could be related to increasing plasma basal PGE_1_ levels, taking into account the anti-inflammatory and vasodilator role of PGE_1_ [11, 39]. There are several possible origins of plasma PGs as a result of the ubiquitous presence of COX enzymes, including muscle, immune cells and platelets; therefore muscle could be a source of PGs during exercise [38]. Regular exercise training *per se* influences the phospholipid fatty acid composition of muscle membranes, but has no effect on the composition of fatty acids stored in triacylglycerol within the muscle [15]. A training season was able to induce changes in the muscle phospholipid composition in such a way as to enhance the capabilities to PGE_1_ synthesis, in accordance with the increased plasma PGE_1_ levels present after 8 weeks of training. The different pattern for plasma PGE_1_ and PGE_2_ in response to training and DHA diet supplementation is not surprising, given the different biosynthesis pathways of these two PGs [30]. PGE_2_ is synthesized from AA (C20:4 Δ5, 8, 11,14) [30], whereas PGE_1_ is synthesized from dihomo-γ-linolenic acid (DGLA) (C20:3 Δ8,11,14), a precursor of AA [30]. The relative differences of plasma PGE_2_ versus PGE_l_ levels is not merely a function of the relative abundance of AA versus DGLA in tissues, but could also be related to the different cellular metabolism of these two fatty acids. Chronic exercise induces changes in the enzyme activities related to eicosanoid metabolism [26]; elongase activity increases but Δ5- and Δ9-desaturase activities have no consistent changes attributed to chronic exercise [26], although Δ5-desaturase was found to be generally lower in muscles of trained rats [14]. Due to the limited activity of Δ5-desaturase, most DGLA is inserted into membrane phospholipids at the same C-2 position as for AA [30]. This picture is in accordance with the effects of a training season on basal plasma PGE_1_ levels enhancing DGLA availability in muscle phospholipids, in order to facilitate PGE_1_ synthesis via an increase in COX-1 and COX-2 muscle activities. It is also in accordance with a lack of effects of exercise on the fatty acid composition of muscle lipids reported by a nutritional intervention [26], because no effects of DHA diet supplementation are observed on the plasma levels of PGE_1_.

Acute exercise increases intramuscular PGE_2_ levels [17], and also increases or does not affect PGE_2_ plasma levels [9], which are attributed to muscle PGE_2_ production. Neither DHA diet supplementation nor acute exercise altered. PGE_2_ plasma levels, but these two factors interact resulting in a significant increase in plasma PGE_2_ levels after acute exercise in the experimental group. PGE_2_ has pro-inflammatory or anti-inflammatory effects depending on location: it enhances LTB4-mediated neutrophil extravasation and tissue injury, but it inhibits the NFκβ signalling pathway via the EP4 receptor, the LPS-induced IL6 release [26], and plays a critical role in the initiation of lipid mediator class switching [32]. PGE_2_ has been evidenced to suppress lymphocyte proliferation and natural killer cell activity, and to inhibit the production of TNFα, IL1, IL6, IL2 and IFNγ [4]. Acute exercise and DHA diet supplementation have synergistic effects against inflammation by enhancing PGE_2_ levels in plasma. These results could reinforce the idea that practising regular exercise results in health benefits and reduces the risk of inflammation related diseases [25].

### Effects on PBMC capabilities to produce lipid mediators and cytokines

It is well established that dietary ω-3 PUFA alters the fatty acid composition of immune cells [36]. This altered fatty acid composition could affect immune cell function [6, 7] and the capability to produce cytokines and lipid mediators. Similarly, training *per se* alters the fatty acid composition of phospholipids and enzyme activities of the eicosanoid metabolism [26]. Protein levels of COX-1 and COX-2 significantly increased in PBMCs after 8 weeks of a training season, mainly in the DHA supplemented group. In addition, this is the first time that an enhancing effect of DHA diet supplementation on COX-2 levels has been described in PBMCs. This could indicate greater capabilities of PBMCs after training and after acute exercise in the DHA supplemented group to transform DGLA into PGE_1_ or AA into PGE_2_, which in fact could increase basal plasma PGE_1_ and PGE_2_ levels; however, the changes in PGE_1_ and PGE_2_ plasma levels are not consistent with these pictures. This suggests that PBMCs are not responsible for plasma PGE_1_ and PGE_2_ levels, even after acute exercise. COX-2 protein levels were significantly higher in the DHA diet supplemented group than in the placebo group, both in basal and post-exercise conditions. These results are in accordance with previous studies, in which an increase in both COX2 activity and protein levels was observed after resistance exercise, and this increase was maintained among 24 h [8]. The COX-2 isoform is inducible and is involved in febrile and inflammatory responses [38], whereas the COX-1 isoform is constitutively expressed in most cells and catalyzes the production of PGs involved in homeostatic control and cell maintenance. Synergistic effects of DHA diet supplementation and acute exercise on PBMC COX-2 levels are not reflected in the rate of PGE_1_ and PGE_2_ production by LPS-stimulated PBMCs. It seems that increased levels of COX-2 have no functional consequences, because DHA diet supplementation decreases the production rate of PGE_1_ and PGE_2_ by LPS-stimulated PBMCs. Discordances between COX protein levels and COX activity have been previously described [8]. Acute exercise transiently increased COX-1 activity in muscle independently of COX-1 protein levels; in contrast, both COX-2 activity and protein levels were elevated with exercise and this rise persists until at least 24 h after resistance exercise [8]. In contrast, DHA diet supplementation did not alter gene expression of NFκβ, COX-2, 15-LOX2, IL1β or 5-LOX in PBMCs, even after acute exercise.

LPS-stimulated PBMCs also synthesize RvD1. Rvs are lipid mediators produced from ω-3 PUFA such as EPA (Resolvin E) and DHA (Resolvin D) [33]. RvD1 is produced from DHA through a pathway initiated by COX-2 [33, 34]. Oil fish diet supplementation has been demonstrated to increase leucocyte capabilities to produce Rvs [33]. RvD1 has pro-resolving and anti-inflammatory properties by inhibiting trans-endothelial migration of neutrophils, preventing their infiltration into sites of inflammation and inhibiting IL1β production [33, 34]. The RvD1 production rate by LPS-stimulated PBMCs was significantly increased after exercise, mainly in the DHA supplemented group. Rvs are synthesized by immune cells after initiation of inflammation in order to contribute to its complete resolution and returning to homeostasis [32]. These results are in accordance with previous studies showing an increase in RvD1 serum levels after acute exercise [22], which could be related in part to the anti-inflammatory effects of regular exercise [25]. LPS-stimulated PBMCs simulate the effects of an infection. In self-limited *Escherchia coli* infections, resolution programs were activated in mice, and RvD1 and RvD5 were elevated [10] in accordance with the effects of LPS activation on RVD1 production rate by PBMCs. The greater capabilities to produce RVD1 by LPS-stimulated PBMCs at post-exercise respect to basal could indicate that post-exercise circulating immune cells are switching in order to resolve inflammation to a greater extent than basal samples. More studies are needed to assess whether other lipid mediators influence the anti-inflammatory effects associated with physical exercise.

LPS stimulates PBMC basal production rate of cytokines such as IL6, IL8, and TNFα [6, 7]. LPS links to TLR4 by activating the NFκβ pathway and inducing an inflammatory response [27], which was evidenced by increased NFκβ activation after LPS-stimulation of PBMCs at 37 °C and 39.5 °C and increased basal rate of IL1b production. Acute exercise also increased active NFκβ in PBMCs, but it was in parallel with a lack of acute exercise effects on the expression of inflammatory genes. Active NFκβ translocates to the nucleus allowing the expression of inflammatory genes but additional stimulus must be operative to enhance the expression of NFκβ, COX-2, 15-LOX2, IL1β and 5-LOX in PBMCs after acute exercise. Acute exercise increased active NFκβ levels priming PBMCs for inflammatory response.

Acute exercise increased PBMC production rate of MCP1 and also primes PBMCs to respond to LPS-stimulation in order to enhance their PGE_1_, PGE_2_, RvD1 and MCP1 production. These results are in accordance with increased levels of TLR4 found in PBMCs after acute exercise, and with the higher rate of cytokine production after LPS-stimulation of post-exercise PBMCs [6, 7]. DHA diet supplementation attenuated the response of acute exercise to stimulation of PBMCs with LPS. The anti-inflammatory effects of DHA-diet supplementation have been described in previous studies [6, 7, 36]. DHA-supplementation attenuates the effects of acute exercise by increasing TLR4 levels in PBMCs [6, 7]. ω-3 PUFA can interfere with TLR4 activation by LPS; this process inhibits signalling components downstream from TLR4 but can also directly prevent NFκβ activation by impeding I-κβ phosphorylation and therefore prevent NFκβ translocation into the nucleus [27].

## Conclusion

In summary, regular training promotes systemic anti-inflammatory and vasodilator effects by increasing PGE_1_. Acute exercise and DHA diet supplementation act synergistically by increasing plasma PGE_2_ and also with anti-inflammatory effects. Acute exercise primes PBMCs to enhance PGE_1_, PGE_2_ and RvD1 production rates in response to LPS, with a higher anti-inflammatory and resolute profile for PBMCs after exercise. The differences between eicosanoid production by PBMCs and plasma eicosanoid levels reinforce the idea that PBMCs are not the main contributors to plasma PGE_2_ and PGE_1_ after acute exercise.

Finally, prescription of regular exercise, similar to football training, could produce anti-inflammatory effects associated to an increase in PGE1 and PGE2 levels. Furthermore, the enhancement of muscular prostaglandins synthesis, probably trough exercise, could subserve these anti-inflammatory and vasodilator effects. In addition, acute exercise and DHA supplementation can act synergistically in the induction of anti-inflammatory response, but also both factors may reduce the inflammatory response induced by pathogen-associated molecular pattern (PAMPs) as LPS.

### Limitation of the study

The experimental procedure was designed to perform exercise at intensity higher than 70 % VO_2_max for more than 50 % duration of the session in order to induce an oxidative stress situation. These conditions were followed by all participants in the study; however, we did not realized any quantification of the effort using portable metabolic carts, activity monitors or video analysis to confirm energy expenditure or movement pattern, and this fact could be the main limitation of the study. Another limitation was low number of participant in each group. Twenty-two athletes, eleven in each group, participate in the trail at the beginning of the study; unfortunately, six athletes left the football team during the trial to join the first and professional team and one broke the anterior cruciate ligament of the knee.

## Abbreviations

AA: arachidonic acid
ANOVA: analysis of variance
BMI: body mass index
COX: cyclooxygenase
DGLA: dihomo-γ-linolenic acid
DHA: docosahexaenoic acid
IFNγ: interferon gamma
IL1: interleukin 1
IL1β: interleukin 1β
IL2: interleukin 2
IL6: Interleukin 6
LOX: lypoxigenase
LPS: lipopolysaccharide
MCP1: monocyte chemotactic protein 1
NFκβ: nuclear factor κβ
PAMPs: pathogen-associated molecular pattern
PBMCs: peripheral blood mononuclear cells increased basal
PGE: prostaglandin E
PPARγ: proliferator-activated receptor
PUFA: polyunsaturated fatty acids
RvD1: resolvine D1
TLR: toll like receptor
TNFα: tumour necrosis factor α
WHR: waist-hip ratio
